# Supplementary material for: Application of Light-Sheet Mesoscopy to Image Host-Pathogen Interactions in Intact Organs
Source: Front Cell Infect Microbiol. 2022 Jun 14;12:903957. doi: 10.3389/fcimb.2022.903957 (PMC9237429; doi:10.3389/fcimb.2022.903957)
Supplement: Supplementary file 1 [file Table_1.docx]

Supplementary Material


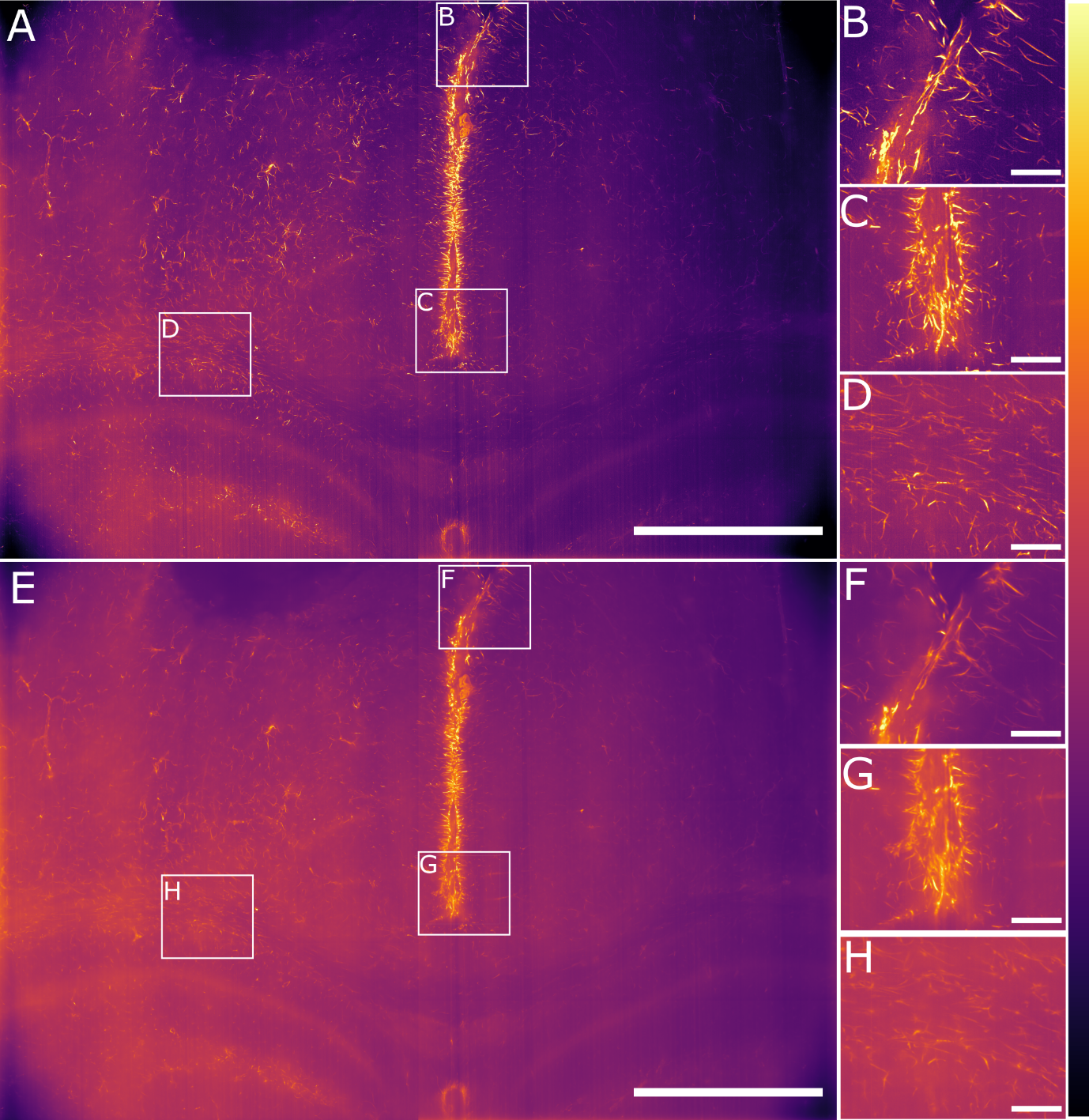


**Supplementary Figure 1.** Comparison between deconvolved and non-deconvolved data shown in Figure 4. The deconvolved data are shown in panels (A-D) while the non-deconvolved corresponding areas are in panels (E-H). The scale bar is respectively (A)-(E) 1000 µm and (B-D)-(F-H) 100 µm. The images were converted to 8-bit values (0-255).
